# Assessing patient safety in a pediatric telemedicine setting: a multi-methods study

**DOI:** 10.1186/s12911-020-1074-7

**Published:** 2020-04-03

**Authors:** Motti Haimi, Shuli Brammli-Greenberg, Orna Baron-Epel, Yehezkel Waisman

**Affiliations:** 1 Clalit Health Services, Digital Health Wing, Central Division, Tel Aviv, Israel; 2 Rappaport Faculty of Medicine, Technion, Haifa, Israel; 3 School of Public Health, Faculty of Social Welfare & Health Sciences, University of Haifa, Haifa, Israel; 4 Clalit Health Services , Sharon- Shomron District, Hadera, Israel; 5 The Emergency Department, Schneider Children’s Medical Center, Petach-Tikvah, Israel; 6 Faculty of Medicine, Tel Aviv University, Tel Aviv, Israel

**Keywords:** Telemedicine, Pediatrics, Patient safety, Appropriateness, Reasonableness, Decision-making

## Abstract

**Background:**

Telemedicine and telephone-triage may compromise patient safety, particularly if urgency is underestimated. We aimed to explore the level of safety of a pediatric telemedicine service, with particular reference to the appropriateness of the medical diagnoses made by the online physicians and the reasonableness of their decisions.

**Methods:**

This retrospective multi-method study investigated the decision-making process of physicians in a pediatric tele-triage service provided in Israel. The first section of the study investigates several measures relating to patient safety in the telemedicine setting. Two physicians reviewed a random sample of 339 parent-physician consultations conducted via a pediatric telemedicine service provided by a healthcare organization during 2014–2017. The consultations were analyzed for factors that may have affected the online physicians’ decisions, with an emphasis on the *appropriateness* of the diagnoses and the *reasonableness* of the decisions. The online physicians’ decisions were also compared to the subsequent outcomes (i.e., parental compliance with the recommendations and medical follow-ups within the healthcare system) after each consultation.

The second section of the study (using a qualitative approach) consisted of interviews with 15 physicians who work in the pediatric telemedicine service, in order to explore their subjective experiences and efforts for assuring patient safety. The physicians were asked about factors that may have affected their reaching an appropriate diagnosis and a reasonable decision while maintaining patient safety.

**Results:**

The first section of the study demonstrates high levels of diagnosis appropriateness (98.5%) and decision reasonableness (92%), as well as low levels of false-positive (2.65%) and false-negative (5.3%), good sensitivity (82.85%), and high specificity (96.15%). A high association between the online decisions and the subsequent outcomes was also observed. The second section of the study presents physicians’ means for ensuring high patient safety – by implementing a range of factors that helped them reach appropriate diagnoses and reasonable decisions.

**Conclusions:**

The results show overall high patient safety in the pediatric tele-triage service that was examined. However, decision makers must strive to implement additional means for further enhancing the clinicians’ ability to reach accurate diagnoses and provide optimal treatments within the tele-triage settings – with the aim of ensuring patient safety.

## What is the key message of your article?

- High levels of patient safety are demonstrated in this pediatric telemedicine service.
- Unique and specific measures are implemented by physicians working in this setting to promote patient safety.
- Analysis shows a positive correspondence between online-physician’s decisions and the “subsequent outcomes” (i.e., parents’ compliance with guidelines and the system’s performance following each consultation).

## What does it add to the existing literature?

- Telemedicine and telephone triage may compromise patient safety, particularly if urgency is underestimated and especially with nurse-tele-triage.
- Previous researches on the safety and quality of tele-triage services have presented conflicting results.
- Unlike most previous studies, this research investigates the decision-making process of physicians, not nurses, in a pediatric telemedicine service.
- This analysis of patient safety in a pediatric tele-triage service incorporated several measures of the physicians’ decision-making process: diagnosis “appropriateness”; decision “reasonableness”; and the physicians’ subjective perspectives.

## What is the impact?

- This study supports previous studies that found telephone triage to be safe for patients.
- The results demonstrate overall high safety of this pediatric telemedicine service with excellent diagnosis appropriateness and good decision reasonableness. In addition to high patient compliance, this service was found to help reduce the workload of GPs and EDs.
- Despite the overall high safety in this pediatric telemedicine-triage service, decision-makers must strive to find additional means for further improving diagnoses accuracy and optimal treatment decisions.

## Background

When geographical distance separates participants, telemedicine may be employed as a means of telecommunications for providing remote diagnostic and therapeutic services. Indeed, telemedicine was originally utilized for providing individuals with healthcare services in rural areas, at home, and in places where medical personnel were not readily available [1]. Unlike regular face-to-face medical encounters, physicians in the telemedicine setting are required to provide a diagnosis without being able to perform a physical examination. Telemedicine has presented numerous advantages and achievements [2], yet this means of medical service poses several difficulties and challenges for physicians [3].

Over the past few years, more and more western countries and large-scale organizations have begun to provide out-of-hours primary healthcare services. For example, the telephone-triage (tele-triage) mechanism operated mainly by nurses, which determines the medical urgency and appropriate type of healthcare required when contacted by patients via the telephone. This mechanism plays an important role in providing accessible, efficient, and safe healthcare [4].

In the emergency tele-triage setting, the decision-making process is complex and stressful, as decisions have to be made within seconds and based on partial, unreliable, and non-visible information and cues. Furthermore, patients vary in their ability to communicate their symptoms coherently – especially when children are involved. Finally, there are no clear-cut criteria for making the decision, exacerbating the difficulty of the decision-making process even more [5, 6].

Different types of tele-triage systems have been established by different healthcare systems. In some countries, tele-triage is performed by physicians, while in others it is managed by nurses or non-clinicians [7–9]. The ultimate telemedicine goal is to provide suitable healthcare services via telecommunication in locations or at times where physical healthcare services are unavailable. As such, the key question is whether such a service can provide equally good or satisfactory diagnostic and management decisions as face-to-face healthcare services [10].

In some cases, tele-triage may compromise patient safety, particularly if time urgency is underestimated by the service provider, rendering the patient without the necessary treatment and within the necessary timeframe [9]. In addition to urgency, patient safety could be compromised for several reasons, especially as telephone medicine eliminates necessary visual cues, and after-hour call systems impose certain barriers that may impact the healthcare provided. Studies show that identifying medical urgency via out-of-hours telephone services is suboptimal [7]. In nurse tele-triage, for example, discrepancies have been reported between diagnoses made via the phone versus face-to-face diagnoses [11–13].

Previous studies have employed a range of methods and concepts for analyzing tele-triage medical decisions and quality. For example, “appropriate referral” to the hospital compared to “under referral” [14], the accuracy of the diagnosis [5], and the appropriateness of the diagnosis [4]. The results show that not only do triage in general, and tele-triage in particular, require fine reasoning skills and decision-making abilities based on sound clinical judgment, but they also depend on contextual factors and on the caregivers’ level of experience [15].

Several studies from recent years have reached conflicting results regarding the safety and quality of tele-triage services. Some studies were pessimistic**,** reporting that patient safety is often compromised due to tele-triage decisions [11], service providers do not always forward the case to the on-call physician when necessary [12], and only few diagnostic and management decisions made during tele-triage consultations provide the same level of healthcare as face-to-face interactions [10].

Some studies on tele-triage services provided by physicians [6] reported that the most common allegation was failed diagnosis (68%) and the most common injury was death (44%). Other studies [8] that investigated tele-triage services provided by nurses presented a correct estimation of urgency in 69% out of the 352 cases examined, with a 19% underestimated level of urgency. The study concluded that tele-triage conducted by nurses may not be safe, having potentially severe consequences for the patient. When high-risk simulated patients were investigated [9], only 46% of the cases were found to be safe. When comparing between decisions made in tele-triage services by clinicians (i.e., physicians or nurses) and non-clinicians [14], the researchers concluded that tele-triage decision-makers should be clinicians, yet that even with clinicians, the safety ratio of the tele-triage decisions should be improved. The triage process was also investigated in a standard pediatric emergency setting [15], which led to the conclusion that in emergency situations, triage decisions are often non-analytic, based mainly on intuition, and guidelines are not used uniformly by all nurses throughout the same tele-triage process.

Other studies on safety in tele-triage services report more encouraging results. Some studies investigated the “appropriateness” of the decisions made in the tele-triage consultation: For example, Blank et al. [16] reviewed studies in which the advice given by telephone was compared to advice that is considered to be appropriate in that given situation (i.e., the “gold standard” of professional advice.) This review found a 44–98% rate of accuracy/appropriateness, with a 75% median. Another study [4] found the majority of decisions to be appropriate, whereby decision appropriateness was found to be positively related to higher consultation quality (including medical knowledge and communication), while high urgency was associated with suboptimal consultation quality**.**

Carrasqueiro et al. [17] reviewed studies in which medical records were examined to determine the adequacy of the advice provided by phone and concluded that tele-triage advice services are reasonably safe. Champagne et al. [18] evaluated the performance of a nurses’ system and found that sensitivity was maximized (96%) when tele-triage decisions related to financial resources. The specificity was 55% and this percentage increased as decisions became more “costly” in terms of risk or availability of scarce resources (i.e., physicians) in emergencies. This finding led the researchers to conclude that tele-triage requires professional judgment in additional to professional knowledge and skills.

In general, it is agreed that in order to improve patient safety and maintain a high quality of healthcare, professional and experienced physicians are needed, as are means for optimizing clinical decision-making [19]. In relation to telemedicine, these insights may be even more crucial, as physicians who provide telemedicine and tele-triage services face unique and additional difficulties and challenges – especially in pediatric telemedicine settings [3].

### Pediatric telemedicine service

In 2009, Clalit Healthcare Services established a unique and much needed pediatric telemedicine service in Israel. Although the service is provided by Clalit, a managed care organization (MCO), this *Clalit Pediatricians Online Service* is actually operated by *Femi Premium* Ltd., who recruits the general and specialist pediatricians and provides the technological infrastructure for operating the service. The service offers remote medical consultations for parents who seek urgent, out-of-hours medical advice about their children (e.g., at evenings, nights, weekends, or holidays, when community healthcare clinics are closed.) The physicians can communicate with the parents by telephone or live video chat. The physicians also have access to the children’s medical records, including their laboratory results and data imaging, as well as information from their previous visits to community clinics, emergency departments (ED), or hospitals.

The service’s main goal is to carry out a tele-triage for deciding which cases are urgent enough to warrant a referral to the ED, and which can be addressed by community doctors during regular working hours. In non-urgent cases, the online pediatricians may also provide parents with medical instructions and digital prescriptions if necessary [20]. To the best of our knowledge, the physician’s decision-making process in a pediatric telemedicine setting has not yet been analyzed or described.

### The aim of the study

The study explored patient safety within a pediatric tele-triage service, based on the diagnosis appropriateness and decision reasonableness of the physicians who provide this service. The study also aimed at examining the physicians’ efforts and methods for maintaining patient safety.

The term *diagnosis appropriateness* is used to describe whether the online physician reached the “correct” presumed diagnosis based solely on the “pure” medical data that was provided during the consultation. This term refers to the appropriateness of only the diagnosis and the associated clinical reasoning (not based on laboratory findings).

The term *decision reasonableness* is used to describe the quality of the therapeutic decision made by the online physician, based on the service’s protocols and the medical and non-medical information provided during online encounter.

The degree of diagnosis appropriateness and decision reasonableness of each medical case examined in this study was determined by the evaluation of external reviewing physicians, who listened to the tele-triage recordings and read the written data from each consultation.

## Methods

This research employed a multi-method study that consists of both quantitative and qualitative approaches, implemented in the two sections of the study. *The first section* presents the analysis of a random sample of 339 telephone consultations conducted by physicians who worked at *Clalit Pediatricians Online Service* during 2014–2017. In this section, several measures of patient safety were analyzed. Using a qualitative approach, *the second section* involves the interviewing of physicians who provided the service, in order to obtain their subjective perspectives about maintaining patient safety in this setting. The physicians were asked about factors that may have impacted their reaching a “correct” diagnosis and deciding on reasonable and appropriate treatment. The use of several approaches helped deepen the understanding of the findings.

### **Section I**

The researchers received a random sample of 1027 recorded telephone consultations that took place during 2014–2017 between online pediatricians and parents who were seeking medical advice for their children. Out of these calls, only those that were considered urgent were included in the study, while calls regarding general consultations, prescriptions or information requests, or follow-up calls were excluded from the study. In the final count, 339 recorded consultations were included – considered to be of urgent nature and having complete documentation (i.e., recorded conversation and written files).

This part of the research included a double-blinded evaluation, whereby two doctors, independently and separately, listened to each telephone conversation and reviewed its respective written summary. The doctors then wrote their medical opinion of the diagnosis, based “purely” on the medical information that was available for each consultation. This information, which was derived from the recordings and documentation, was presented to each of the two doctors-reviewers in a detailed database table, including medical symptoms, severity of symptoms (mild/ moderate/severe), duration of symptoms, number of times the online service had been used by the same patient and for the same symptoms, number of in-person visits to the community clinic regarding these symptoms, additional diseases, and the physician’s adherence to the service’s medical guidelines (i.e., protocols).

*Adherence to protocols* was defined as whether the online-physician complied with the instructions that appeared in the protocols that had been prepared by the online-service supervisors for specific medical problems (e.g., Infants with head trauma and under the age of 12 months must be referred to the ED.)

Potential non-medical factors that could be obtained from the conversations or written summaries of each consultation, as well as their possible effect on the diagnosis and on the final therapeutic decision, were also evaluated. These included variables regarding the child, parents, place of residence, physician, shift, and interaction between the physician and the parent – such as the physician’s impression of the parents’ health literacy and whether a shared decision making (SDM) strategy had been employed. Neither reviewer had access to the input provided by the other reviewer.

The use of SDM was determined based on the following criteria: Both the physician and the patient (or in this case, the parent) were involved in the treatment decision-making process; both the physician and the parent shared information; both made an effort to participate in the decision-making process by expressing treatment preferences, and both agreed on the treatment that was decided upon [21].

Each consultation and its final decision were analyzed in order to identify and categorize the dependent variables. These include the primary decision, secondary decisions, diagnosis appropriateness, and decision reasonableness.

#### Primary (main) decision

This primary outcome was related to the physician’s main decision whether to refer the patient to the ED or not.

#### Secondary (sub) decision

In cases where the patients were not referred to the ED, the online physician’s secondary decisions were examined according to two main categories:

- Follow-up only (i.e. “wait and see”);
- More “active” decisions: Intervention (providing treatment such as sending a digital prescription or other instructions), or determining that additional information is needed (e.g., arranging a video conversation, consulting with the attending physician, suggesting a follow-up conversation at a later time, or recommending going to an emergency center in the community).

#### Diagnosis appropriateness

For each consultation, the two evaluating physicians rated the online physician’s diagnosis as either appropriate or inappropriate (i.e., Appropriate: yes / no), based solely on the medical data (i.e., the symptoms presented during the consultation) that was available to the online physician, in the most objective manner. This term refers to the appropriateness/“accuracy” of the diagnosis and associated clinical reasoning (but not compared to laboratory findings and additional medical data that may have been achieved later.)

#### Decision reasonableness

For each consultation, the two evaluating physicians rated the online physician’s therapeutic decision reached as either reasonable or unreasonable (i.e., Reasonable: yes / no) with regards to the specific medical problem, based on the online service’s clinical protocols and all available information (medical and non-medical).

Diagnosis appropriateness and decision reasonableness were considered factors that are associated with patient safety in the telemedicine setting and were categorized as dichotomous variables (yes / no), i.e. whether the diagnosis was appropriate/inappropriate and whether the decision was reasonable/unreasonable.

This dichotomous evaluation of the diagnosis appropriateness and decision reasonableness of each case was based on the method developed by Champagne et al. [18].

The two independent evaluating physicians in our study included a pediatric specialist and a pediatric emergency medicine specialist, both highly experienced in telemedicine. In cases where there were differences between the evaluations of the two physicians, a third doctor (a senior pediatrician)– was asked to review the telephone conversation and written records of the case in question and judge the diagnosis appropriateness and decision reasonableness. As such, each evaluation was determined based on the agreement of two doctors. While the reviewers’ evaluations may be perceived as subjective, these evaluating physicians were instructed to maintain maximum objectivity throughout their evaluations, basing their decisions solely on the medical data provided which was organized and presented to them in a quantitative manner via a computerized database.

Primary decisions were considered *false negative* (FN) in cases where the online-physician did not refer the patient to the ED but both evaluating physicians wrote that the child should have been referred (i.e., the online physician provided suboptimal intervention and did not refer the patient to the ED when necessary); primary decisions were considered *false positive* (FP) when the online physician referred the child to the ED but the two evaluating physicians agreed that the child should not have been referred (i.e., the patient was referred to the ED needlessly).

Sensitivity, specificity, positive predictive value (PPV), and negative predictive value (NPV) were also calculated. These terms are explained as follows:

*Sensitivity* is defined as the proportion of people **with** the disease who will have a **positive** result (percentage of true positives). In our study, this term refers to the percentage of children that were actually referred to the ED by the online physician, among all those who should have been referred to the ED (according to the two reviewing physicians).

*Specificity* is defined as the proportion of people **without** the disease who will show a **negative** result (i.e., the percentage of true negatives). In our study, this term refers to the percentage of children who were not referred to the ED by the online physician out of all the cases that should not have been referred to the ED according to the two reviewing physicians.

*PPV* is the probability that following a positive test result, the individual will truly have that specific disease. In this study, this term refers to the percentage of children who were correctly and necessarily referred to the ED (according to the reviewers) out of all those who were actually referred to the ED by the online physicians.

Finally*, NPV* is the probability that following a negative test result, the individual will truly not have the specific disease. In our study, this term refers to the percentage of children who should not have to be referred to the ED (according to the reviewers) out of all those who were not referred to the ED by the online physicians.

Further explanations for these values appear in reference [22].

In addition, *subsequent outcomes* were examined based on data presented in Clalit’s computerized database (which includes data from all clinics and hospitals in Israel). This data enabled the examination of the final outcome for each patient whose parents had contacted the online healthcare service, including follow-up visits, diagnoses achieved by additional (community) physicians, visits to the ED, and parents’ compliance with the physician’s instructions after the online consultation. For children who were referred to the ED, we checked whether or not they actually visited the ED and the outcome (and the diagnosis reached); for cases that were not referred to the ED, we checked whether or not they followed up with their community physicians within 2 days of the online consultation (and the diagnosis reached during those visits).

All necessary approvals from the Ethics Committees of Clalit Healthcare Services and the University of Haifa were obtained (numbers 0031-16COM2, and 458/16 respectively).

#### Statistical analysis

SPSS version 24 was used to conduct statistical analysis of the quantitative data, with continuous variables presented in the form of means, standard deviations, and medians, and categorical variables presented in percentages.

Each of the following outcomes was analyzed separately:

*Primary decision* (ED referral – yes/no); *Secondary decision* for those not referred to ED (“wait & see” or interventions such as sending digital prescriptions or further investigation via video chat, etc.); *Diagnosis appropriateness* (yes/no); *Decision reasonableness* (yes/no).

For each outcome, differences in medical and non-medical characteristics were compared using independent t-tests or Mann-Whitney tests for the continuous variables, and Chi-square tests for the categorical variables. The significant variables in the univariate analysis were entered into a multivariate logistic regression to examine which variables are independently correlated to the outcomes. Odds Ratio (OR) with 95% Confidence Interval (CI) are also shown. In addition, Kappa analysis was used to examine the level of agreement between the two reviewing doctors regarding decision reasonableness. *P* < 0.05 was considered statistically significant.

### **Section II**

In this qualitative section of the study we interviewed 15 physicians who had worked at the *Clalit Pediatricians Online Service* for the last five years. In order to analyze their input and identify themes, Thematic Analysis was used – a flexible research tool that provides a rich account of qualitative data. The aim was to reflect and describe the physicians’ experiences during their shifts at the pediatric telemedicine service, especially regarding their efforts and methods for maintaining patient safety. The themes were identified from the participants’ responses.

In order to assess whether the themes accurately reflect the original data, they were evaluated against the original transcriptions, thereby ensuring a consistent progression. The analytical procedure included several stages. First, one researcher identified and labeled themes from each of the 15 transcribed interviews. Next, the researchers as a group discussed the identified themes, to ensure agreement between all the researchers of this paper. Finally, the themes were integrated into a table that reflected the insights of the group as a whole.

A semi-structured qualitative study (SSQS) methodology was used to obtain rich and direct data that would reflect the physicians’ subjective experiences. Using an interview questionnaire, the physicians were asked about the difficulties they faced when working as an online physician, and about factors that affected their ability to provide a correct diagnosis, make reasonable decisions, and decide about appropriate treatment – all while maintaining patient safety**.** The physicians were also asked about their professional training and experience, perceptions and opinions about the ability to maintain patient safety in this tele-triage setting, and suggestions they may have for increasing diagnosis appropriateness and decision reasonableness [3].

## Results

### **Section I**

#### Medical and general characteristics

Table 1 presents medical factors and non-medical characteristics relating to the patient, family, doctor, and shift.

**Table 1:** General and Medical Characteristics (quantitative study)

	Range	Mean	Median	SD	Other	
**Patients**						
Age (years)	0.01–17.3	3.43	1.70	4.04	–	
Gender	–	–	–	–	**Boys**- 171 (50.4%);	**Girls**-168 (49.6%)
Background	–	–	–	–	Healthy −92.3%	WB/Asthma- 4.2%
**Parents/ Family**	**Range**	**Mean**	**Median**	**SD**	**Other**	
Religion	**Jewish**-326 (96.2%)				**Non-Jewish**- 13 (3.8%)	
Gender of Parent who Called	**Mothers**-267 (78.8%);		**Father**s-65 (19.2%);		**Both**-5 (1.5%);	The **child**-1 (0.3%)
**Place of Residence**						
Type	**Small place** (town, village, kibbutz)-119 (35.1%)		**City**-202 (59.6%)			**Missing**-18 (5.3%)
Center/Periphery	**Center**-196 (57.8%),		**Periphery, North**-67 (19.8%)	**Periphery, South**-58 (17.1%)		Missing-18 (5.3%)
**Doctors**						
Age (years)	**40–50**: 78 (23%); **50–60**: 127 (37.5%); **> 60**: 134 (39.5%)					
Gender	**Male**- 240 (70.8%);			**Female** − 99 (29.2%)		
Religion	**Jewish**- 222(65.5%); **Muslim**-86 (25.4%); **Christian**- 31 (9.1%)					
Country of Birth	**Israel**-206 (60.8%); **Not Israel-**133 (39.2%)					
Medical school	**Israel**-150 (44.2%); **Not Israel**-189 (55.78%)					
Specialty type	**General Pediatrics**-298 (87.9%)			**Sub-specialty**-41 (12.1%)		
**Shift**	**Range**	**Mean**	**Median**	**SD**		
Call duration (minutes)	1.18–14.44	3.30	3.13	1.57		
Time of Shift	**Evening** (20.00–24.00)-161 (47.5%)		**Night** (24.00–06.00)-54 (15.9%);			**Day** (06.00–20.00)-114 (33.6%)
Day of Shift	**Weekday**-165 (48.7%)		**Friday**-57(16.8%)			**Sabbath**/holiday-117(34.5%)
Conversation type	**Phone**-330 (97.3%)		**Video**- 6 (1.8%)			**Phone & Photos**- 3 (0.9%)
**Medical Factors**						
Background diseases^**a**^	**No disease**-313 (92.3%)			Wheezing Bronchitis/ Asthma −14 (4.12%)		
Duration of disease	**Several minutes-**10 (2.9%)		**Several hours** (< 24)-208 (61.4%)			**Several days**- 121 (35.6%)
Severity of disease	**Mild-**134 (39.5%)		**Moderate-**190 (56%)			**Severe**-15 (4.4%)
Previous doctor’s visit	**Yes-**77 (22.7%)			**No**-262 (77.3%)		
Times called service	**1 time**-332 (97.9%)			2 times −7 (2.1%)		
Length of conversation (min)	**Range**:1.18–14.54		**Mean**: 3.30			**Median**:3.13
Correspondence to protocols	**Yes-**321 (94.7%)			**No**-18 (5.3%)		

^**a**^ Other diseases (e.g., celiac, autism, ADHD, GER, SVT, Hydronephrosis) - very low prevalence

#### **Dependent variables**

Table 2 presents the dependent variables, including the primary decision, secondary decisions, diagnosis appropriateness and decision reasonableness.

**Table 2:** Summary of Dependent Variables ^a^

Variable	Description			
**Primary Decision** (*n* = 339)	1. **Referred to the ED**: **96** consultations (**28.3%**)		2. **Not referred to the ED**: **243** consultations (**71.7%**)	
**Secondary Decision**^b^ (*n* = 243)	1. **Wait & see:** – Observe the child and visit the community physician the following day: **181** (**74.5%**) consultations.		2. **Active decisions:**^**e**^ **Intervention**: **30** (**12.5%**) consultations (such as sending a digital prescription to the parents.) **Further inquiry**: **32** (**13%**) consultations (using a video, sending photos, visiting the community medical service, arranging an additional conversation, or providing a referral to the ED in case symptoms worsen.)	
**Diagnosis Appropriateness**	1. In **334** (**98.5%)** of the cases, the diagnoses were considered **appropriate** by the two reviewer doctors.		2. In **5** (**1.5%)** of the cases, the diagnoses were considered **inappropriate** by the two reviewing doctors.	
**Decision Reasonableness**	**First stage**^**c**^	In **310** of the consultations, the physicians’ decisions were considered **reasonable** by the two reviewer doctors.	In **17** consultations, the two reviewer doctors agreed that the decisions were **unreasonable**.	In only **12** cases, one reviewer deemed the decision unreasonable, but the other did not.
	**Second stage**^**d**^	In **312 (92%)** of the consultations, the physicians’ decisions were considered **reasonable** by the two reviewer doctors.		In **27 (8%)** of the consultations, the physicians’ decisions were considered **unreasonable** by the two reviewer doctors.

^a^ Descriptive data

^b^ Those that were not referred to ED

^c^ **First stage**: After the evaluation of the two independent reviewer doctors, their input on reasonableness and appropriateness for 12 cases was not uniform. As such, a third independent doctor was asked to review these 12 case, and his decision was final (i.e., two out three doctors’ reviews)

^d^**Second stage**: After the evaluation of the third reviewer, 10 of these 12 undecided cases (from the first stage) were labeled as unreasonable decisions

^e^**Active decisions**: Intervention - providing a treatment (e.g., sending a digital prescription or other instructions); or determining that additional information is needed (e.g., inviting to a video conversation, consulting with the attending physician, suggesting an additional follow-up conversation, recommending going to a community emergency center)

#### Diagnosis appropriateness

In 334 of the cases (98.5%), diagnoses were considered appropriate (“accurate”) by two of the reviewing doctors.

#### Decision reasonableness

In 312 of the consultations (92%), the physicians’ decisions were considered reasonable after comparing the online physicians’ decisions to the reviewers’ opinions.

Calculations of *sensitivity*, *specificity*, *FP*, and *FN* resulted in 82.85, 96.15, 2.65, and 5.3% respectively.

*PPV* = 90.62% and *NPV* = 92.59% (as presented in Tables 3 and 4).

**Table 3:** Decision Reasonableness and ED Referral

Reasonable Decision * ED referral Cross tabulation			
	ED referral		Total
	Yes	No	
**Reasonable Decision**			
Yes			
Count	87 [**TP**]	225 [**TN**]	312
% within Reasonable Decision	27.9%	72.1%	100.0%
% within ED referral	90.6%	92.6%	92.0%
No			
Count	9 [**FP**]	18 [**FN**]	27
% within Reasonable Decision	33.3%	66.7%	100.0%
% within ED referral	9.4%	7.4%	8.0%
Total			
Count	**96**	**243**	**339**
% within Reasonable Decision	28.3%	71.7%	100.0%
% within ED referral	100.0%	100.0%	100.0%

**Table 4:** False Positive and False Negative Calculations

	Should have been referred to ED	Should not have been referred to ED
**ED Referral, Yes (by the online physician)**	**87 TP**	**9 FP**
**ED Referral, No (by the online physician)**	**18 FN**	**225 TN**

**Sensitivity =** TP/ (TP + FN) = 87/105 = 82.85%

**Specificity** = TN/ (FP + TN) = 225/234 = 96.1%

**PPV** = TP/ (TP + FP) = 87/ (87 + 9) = 90.62%

**NPV** = TN/ (TN + FN) = 225/ (225 + 18) = 92.59%

Kappa analysis showed a high level of agreement between the two evaluating physicians in the study regarding decision reasonableness: 0.72, 95% CI (0.57–0.87).

#### Effect of medical factors on the dependent variables

Severity of the disease was the only factor that was found to significantly correlate (*p*, < 0.001) with the primary decision (whether to refer the patient to the ED), whereby problems diagnosed as “severe” received a 100% referral rate to the ED, compared to only a 3.7% referral rate to ED in cases of “mild severity” diagnoses (Supplemental Table 1).

Adherence to protocols was the only factor that was found to significantly correlate (*p* < 0.001) with decision reasonableness. When adherence to protocol was maintained, 95% of the consultations ended with a reasonable decision, compared to only 38.9% reasonable decisions when there was no adherence to protocols (Supplemental Table 2).

Supplemental Table 3 shows factors which are significantly correlated to the secondary decisions including the child’s age (older age categories were associated with more “active” secondary decisions; *p* = 0.007), gender of the child (females were associated with more “active” decisions; *p* = 0.012), and previous doctor’s visit (when the child had not previously visited their regular physician, the online physician’s decisions were more “active”; *p* = 0.038).

#### Effect of non-medical factors on dependent variables

The main non-medical factors which were found to be significant in multivariate analyses were previously reported by us [23]. As summarized in Supplemental Table 4, the results relating to the primary decision show that higher rates of ED referrals significantly correlate with a number of non-medical factors, including place of residency (families living in the northern periphery), doctor’s gender (female physicians), doctor’s specialty (general pediatricians without a sub-specialty), and whether SDM with the parent had been employed (higher rates of ED referrals were observed in cases where SDM was employed).

Moreover, the results show the following non-medical factors to be significantly associated with the secondary decisions: the child’s gender (more “active” decisions with females), the applying parent’s gender (more “active” decisions with fathers), previous visits to the community doctor (more “active” decisions with no previous visits), and whether SDM had been employed (more “active” decisions with SDM).

Finally, the main non-medical factors that were significantly associated with decision reasonableness were the doctor’s age (less reasonable decisions were observed among the older age group) and whether SDM was used (less reasonable decisions were observed when SDM had been employed).

***Table*** ***5*** compares between the primary decision made by the online physician (whether to refer to the ED or not) and the subsequent outcomes, including parents’ compliance and subsequent face-to-face medical encounters. For each online consultation, the researchers also examined the data from the 2 days following the telephone consultation – provided by Clalit, including files from hospitals and community clinics.

**Table 5:** Subsequent Outcomes

Telemedicine Physician’s Primary Decision		Subsequent Outcomes^**a**^			
**The patient was** **not** **referred to the ED**		**Visited a physician within the next 2 days**	**Did not visit a physician within the next 2 days**		**Visited the ED despite having no referral**
	**231**^**b**^ [~ 71.5%]	**122** [~ 37.7%] **Not referred to the ED**	**104** [~ 32.2%]		**4** [~ 1.2%]
		**1** [~ 0.3%] **Referred to ED by the face-to-face physician (and released)**			
**The patient was referred to the ED**		**Visited the ED and was discharged**	**Visited the ED and was admitted**	**Did not visit the ED despite a referral **…	
				**29** [8.9%]	
				**but visited a physician in the community**	**and did not visit physician in the community**
	**92** [~ 28.4%]	**51** [~ 15.7%]	**12** [~ 3.7%]	**14** [~ 4.3%]	**15** [~ 4.6%]
**Total**	***323*** [100%]				
**Missing**	**16**				

^**a**^ Parents’ compliance with the instructions provided by the online physician and follow-up face-to-face medical consultations in the community

^**b**^ In most consultations (231/323 = ~ 71.5%), the online physician provided the parents with comprehensive explanations and instructions and did not refer the child to the ED.

In most consultations (231/323 = ~ 72%), the online physicians provided the contacting parents with comprehensive explanations and instructions, and did not refer the child to the ED. In only 28% of the consultations did the physicians refer the patients to the ED. This finding may indicate the possible advantage of saving of resources when providing such a service.

Among those who were not referred to the ED by the online physician (*n* = 231):

- The vast majority of parents accepted the physicians’ recommendations and did not go to the ED at their own initiative; approximately half (*n* = 123; 52%) went to their community doctor for a follow-up within 2 days of the online consultation. Only one patient was referred by the community physician to the ED and was eventually discharged – suggesting that the online physician had made a good clinical decision.
- Of all the patients who contacted the online service, 45% (*n* = 104) did not need to go to the community clinic for treatment (implying that the treatment or advice they received from the online physician was helpful).
- Only 1.7% (4 cases) went to the ED at their own initiative, without a referral from the online physician. (In such cases, patients are required to pay for the visit themselves as this cost is not covered by their medical insurance through the MCO.) All four were discharged.
- As shown in Tables 3, 4 and 5, the TN value was very high (225 cases) and the FN value was very low (only 18 cases).

Among those who were referred to the ED by the online physician (*n* = 92):

- Of all the children referred to the ED, 68% (*n* = 63) actually visited the ED and were examined there.
- Of all those referred to the ED, 55% (*n* = 51) were released after being examined.
- Of all those referred to the ED, 13% (*n* = 12) were hospitalized.
- Of all those referred to the ED, 31% (*n* = 29) did not go to the ED. Approximately half of them (*n* = 14; ~ 15%) preferred going to the community physician over the next 2 days following the online consultation. The remainder (*n* = 15; ~ 15%) did not visit the ED and did not visit their community physician.
- Even when considering the assumption that those who did not visit the ED despite being referred showed clinical improvement (allowing them to wait for the community physician or not going for a follow up at all), the overall TP value was high (68%).

### **Section II**

Fifteen physicians were interviewed for this research. Table 6 presents the characteristics of these physicians, and Supplemental Table 5 presents the quotes from the interviews with those physicians.

**Table 6:** Physicians’ characteristics (qualitative study)

Main Characteristics of Physicians	
**Gender**	**Males**-9, **Females**-6
**Age (years)**	**Age groups: 40–50**: 6; **50–60**: 7; **> 60**: 2
	**Range:** 42–67; **Mean:** 52.33; **Median:** 53
**Religion**	**Jewish**: 11; **Christian**: 1;**Muslim**: 3
**Place of birth**	**Israel**: 11; **Not Israel**: 4 (Ukraine-1, Russia-1, USA-1, Argentine-1)
**Place of medical studies**	**Israel**: 10; **Not Israel**: 5 (Italy-2, Russia-1, Argentine-1, Ukraine-1)
**Major place of work**	**Community:** 11; **Hospital:** 4
**Specialty (general vs. specialized)**	**General pediatricians:** 8; **Specialized pediatricians**: 7
**Experience as Pediatrician (years)**	**Range:** 4–30; **Mean:** 19.13; **Median:** 18.46
**Experience in Telemedicine (years)**	**Range:** 0.5–9; **Mean:** 4.76; **Median:** 5
**Active in Pediatric telemedicine**	**Yes:** 11; **No:** 3; **Other:**1 (also active in other telemedicine service)

The following themes were extracted from the physicians’ interviews regarding their efforts and methods for making an appropriate diagnosis and reaching a reasonable decision during online consultations – while maintaining patient safety.

1. ***Use of intuition:*** Many doctors stated that they used their intuition in the diagnostic process, and often applied their intuition about the parents. Some felt that due to the difficulty in assessing the patient’s condition without a physical examination, their intuition played a greater role. *For example, “You learn to rely on your intuition … whether you feel that the parents understand what you are saying, or that in this case, your instructions won’t help.”*
2. ***Experience –*** Physicians often employ their experience to improve diagnostic capabilities. Most doctors feel that their clinical experience in pediatrics in general, and in telemedicine, in particular, helps them with diagnostics and decision making, whereby the more experience they have in telemedicine, the more confident they become. For example, “*During my first few days at work, I was afraid I would miss things or that there would be problems. After a while, however, I began to work with more confidence and less stress.”*
3. ***Use of rules of thumb and protocols*** – Many physicians stated that they used rules of thumb in their decision-making process. Most also made use of the protocols that were available for specific scenarios. They felt that this helped them maintain patient safety. For example, *“I use some rules of thumb. For instance, if a young boy is able to jump around, then he does not have appendicitis.”*
  They were also aware of possible cognitive biases. *“An example of cognitive bias is when there is an exceptional case – after which everyone is much stricter with themselves...”*
4. ***Making shared decisions with the parents*** – Some doctors reported discussing their thoughts about the diagnostic procedure and possible treatment options with the child’s parents. For example, *“I used to share my decision-making process with the parents. If there were several options, I would let the parents decide. In such a case, I depend on them.”*
5. ***Considering non-medical factors*** – Most of the physicians interviewed confirmed that they take non-medical factors into account in their decision-making process, in addition to medical factors. The most prominent factors are the doctor’s impression of the parents, including their level of understanding, level of anxiety, health literacy, and the assurance that the parents will act appropriately if the child’s condition worsens. For example, *“In addition to medical factors, the parents’ tone of voice and level of stress may affect my decision – even if it seems to be a simple diagnosis … Language is also a factor. For example, new immigrants do not always understand me, and I am therefore more prone to sending them to the ED …”*
  An additional important non-medical factor was the accessibility of the family to medical services. For example, *“Aside from the medical condition, the patient’s place of residency is also important. Living far from a medical care facility is a factor, and I will be more likely to consider an ED referral. In such cases, I also ask more questions about the availability of the doctor nearby.*”
6. ***Additional tools*** – In cases of diagnostic uncertainty, physicians may arrange a video chat with the parents, ask them to send digital photos, or arrange a follow-up call a few hours later. A few physicians (generally those who are younger and less experienced) may choose to consult the senior attending physician. For example, *“Despite the difficulty making the decision, pictures and videos often compensate for the lack of a physical examination…”*

In summary, despite difficulties reported by online physicians [3], many of the physicians interviewed in this study reported having general positive experiences with their telephone assessments and feeling that they are able to conduct thorough assessments and make appropriate treatment decisions.

*..."I feel relatively confident and made professional decisions."*

## Discussion

The aim of telephone triage is to ensure that patients with the greatest need for immediate care will receive treatment as quickly as possible, while those with less immediate needs are treated as resources allow [24]. These services help reduce the burden on GPs and EDs, particularly through the reduction of unnecessary or avoidable visits to clinics and hospitals [25].

Despite the well-documented benefits of telephone triage and disease management, these services involve numerous medical, technological, and organizational challenges [3, 26] with regards to their operation and monitoring, as well as service accessibility and quality [27, 28]. Moreover, the complexity and sheer volume of medically related telephone communications may leave patients vulnerable to errors and clinicians vulnerable to malpractice claims [29–31].

As previously mentioned, recent studies on the safety and quality of tele-triage services have reached conflicting results. Leprohon and Patel [5] used the method developed by Champagne [18], which is similar to that employed in this study: A group of experts were presented with the final diagnoses of patients and the information obtained throughout the nurse-patient interactions. They were then asked to reach a consensus about the optimal decision for each telephone consultation. These decisions were then compared with the nurses’ decisions, to determine the accuracy of each decision. The decision was considered *accurate* when both decisions (made by the nurse and the experts) were the same, *FN* in cases where the nurse had not provided sufficient resources (i.e., suboptimal intervention), and *FP* in cases where the nurse had provided more resources than necessary. The researchers found that in high-urgency situations, heuristic rules based on symptoms were used and the related decisions were mostly accurate. With the increase in problem complexity, however, the decisions were more often inaccurate. In moderate-to-low urgency conditions, alternate strategies were used, where contextual knowledge of the situations was employed to identify the needs of the patients and to find the best plan of action to meet these needs, in turn resulting in more accurate decisions.

Using quantitative and qualitative approaches, the present study investigated several measures relating to patient safety in a telemedicine setting, as well as the subjective experiences and efforts of the online physicians and the means they employ to maintain patient safety. The first section of this study demonstrated overall high safety in the pediatric telemedicine setting, with excellent diagnosis appropriateness and decision reasonableness. Low rates of FP and FN and high sensitivity and specificity were also demonstrated. Moreover, when comparing the treatment decision to the subsequent outcomes (including parental compliance with the online physician’s recommendations and later in-person visits), the TN value is very high, the FN is very low, and the TP is impressive. These findings and conclusions resemble those described in previous studies [4, 5, 18], and mainly by Blank [16] and Carrasqueiro [17].

There are a number of possible explanations for the high rate of diagnosis appropriateness. First, the problems presented by the parents were usually straightforward and the physicians were experienced pediatricians (some with sub-specialties). Second, the online physicians were experienced in providing telemedicine services and employed videos chats and additional methods in cases of uncertainty. These explanations are in line with our findings from reviewing 339 consultations and diagnoses in Section I and with Theme 2 (experience) and Theme 6 (use of additional tools) in Section II.

Possible explanations for high decision reasonableness include the following: Most physicians use non-medical factors in addition to the standard medical data, thereby “tailoring” the best treatment solutions to the specific parent and child, time and place. Moreover, most use available protocols that present the recommended treatment for specific situations. This hypothesis can be reached from Theme 3 (use of rules of thumb and protocols), Theme 4 (shared decision making with the parents), and Theme 5 (considering non-medical factors), as well as from the literature [23].

In Israel, when patients are referred to the ED by a physician, the MCO (Clalit, in this case) covers the costs of their admission to the hospital (for a physical examination in the ED, and for the hospitalization if the patient is admitted). When patients visit the ED without a referral from a physician, they are required to cover these costs themselves. For this reason, patients who wish to take their child to the ED, first try to receive a referral from their MCO (via a physician in the community or an online- physician as seen in our study).

In this study, most parents who contacted the online service complied with the instructions and recommendations of the online physician: More than two-thirds of those referred to the ED complied with the physician’s recommendation to go to the ED, and 45% of those who were not referred to the ED did not even need to visit their community physician at a later time – thereby implying good patient compliance and reduced burden on GPs and EDs. These findings correspond with those of Fry [25] and others [26, 27].

Moreover, as the online consultations examined in this study succeeded in providing appropriate care for most of the children, without needing to refer them to the ED, this teleconsultation service helped save resources for both the MCO and the hospitals, as previously described in the literature [24, 27].

As shown in the Section II of the study, the physicians working at the pediatric telemedicine service who participated in this study place an emphasis on ensuring patient safety. To do so, they have developed and employed several techniques to help them reach appropriate diagnoses and reasonable decisions. (This is particularly important in tele-triage, where the physician is unable to perform a physical examination in person.) These techniques include use of intuition, experience, rules of thumb and protocols, sharing their thoughts and dilemmas with parents, considering non-medical factors, and employing video conversations and additional means of communication.

The use of intuition is also reflected in the physicians’ use of other non-medical factors such as considering the parents’ tone of voice or degree of anxiety. These efforts have succeeded in achieving overall high safety, with excellent diagnosis appropriateness and good decision reasonableness – in line with other studies that conclude that tele-triage consultation services are reasonably safe [4, 16–19].

When making a shared decision together with the parents, the physicians who were interviewed for this study also considered the parents’ preferences and their level of uncertainty – even at the cost of referring more patients to the ED, making more “active” secondary decisions, or making less “reasonable” decisions. In some cases, some decisions may be considered “unreasonable”, yet are appropriate to the patients’ (or parents’) specific circumstances and preferences [23].

Putting patients’ safety first can indeed be seen by the fact that although the pediatric telemedicine service described here is offered free-of-cost to patients of the Clalit, the doctors are encouraged to make independent decisions, regardless of economic considerations such as saving money for the MCO.

### Strengths and limitations

This study on a pediatric telemedicine service provides estimates of the safety of tele- triage in out-of-hours healthcare. Patient safety in this study was analyzed using measures of diagnostic appropriateness and decision reasonableness, and by evaluating the subjective perspectives of the online physicians – using quantitative and qualitative methods. The data provided about what actually happens with the patients following their online consultations further strengthens the finding of this study.

Nevertheless, some limitations may exist, in particular regarding the subjectivity and potential for bias in any retrospective review (conducted by two reviewing doctors) of others (the online physicians) who made judgment calls in the past. To increase objectivity, a third independent reviewer was asked to reach a consensus in cases when the two reviewers arrived at different decisions.

When evaluating the subsequent outcomes of parents and the healthcare system following the recommendations of the telemedicine physicians, 16 files were unavailable (some of these patients had become members of a different MCO), and as such we were unable to access the medical records of those patients children. While this missing information may have affected our conclusions based on the subsequent outcomes, there was no effect on our conclusions regarding the diagnosis appropriateness and decision reasonableness – as the recordings of all the online consultations were available for the two reviewing doctors.

## Conclusions

The findings of this study support previous studies that found tele-triage to be safe for patients. Overall, the results demonstrate high levels of patient safety following pediatric telemedicine service, with excellent diagnosis appropriateness and good decision reasonableness, in addition to high patient compliance with recommendations and reduced GP and ED burden. Policy makers and healthcare managers should consider these results when implementing tele-triage in their institution, while recognizing and addressing potential challenges and obstacles that exist in the telemedicine setting and providing clinicians with specific training and knowledge for conducting effective and responsible tele-triage.

## Supplementary information

**Additional file 1: Supplemental Table 1**. Medical Factors and ED Referral.

**Additional file 2: Supplemental Table 2**. Medical Factors and Decision Reasonableness.

**Additional file 3: Supplemental Table 3**. Medical factors and Secondary Decisions.

**Additional file 4 :Supplemental Table 4**. Significant Non-medical factors- multivariate analyses.

**Additional file 5: Supplemental Table 5**. Quotes from Interviews with Physicians by Themes.

## Data Availability

All data generated or analyzed during this study are included in this published article and supplementary information files.

## Abbreviations

CI: Confidence Interval
ED: Emergency Department
FN: False Negative
FP: False Positive
GP: General Practitioner
MCO: Managed Care Organization
NPV: Negative Predictive Value
OR: Odds Ratio
PPV: Positive Predictive Value
SDM: Shared Decision Making
SSQS: Semi-structured Qualitative Study
TN: True Negative
TP: True Positive
