# Supplementary material for: Assessing patient safety in a pediatric telemedicine setting: a multi-methods study
Source: BMC Med Inform Decis Mak. 2020 Apr 3;20:63. doi: 10.1186/s12911-020-1074-7 (PMC7126468; doi:10.1186/s12911-020-1074-7)
Supplement: Supplementary file 1 — Additional file 1: Supplemental Table 1. Medical Factors and ED Referral. [file 12911_2020_1074_MOESM1_ESM.docx]

**Supplemental Table 1: Medical Factors and ED Referral ***

|  |  | **Referral to the ED** | |  |  |
| --- | --- | --- | --- | --- | --- |
| **p-value** | **OR 95%CI** | **Yes**  **N=96** | **No**  **N=243** | **Total**  **339** |  |
|  |  |  |  |  | **Age** |
|  | Ref | 28 (22.6) | 96 (77.4) | 124 (36.6) | <=1 |
| 0.180 | 1.46 (0.84-2.56) | 41 (29.9) | 96 (70.1) | 137 (40.4) | 1-5 |
| 0.259 | 1.55 (0.73-3.31) | 14 (31.1) | 31 (68.9) | 45 (13.3) | 5-10 |
| 0.054 | 2.23 (0.99-5.04) | 13 (39.4) | 20 (60.6) | 33 )9.7) | >10 |
|  |  |  |  |  | **Gender** |
| 0.535 | Ref | 51 (29.8) | 120 (70.2) | 171 (50.4) | Male |
|  | 1.16 (0.72-1.87) | 45 (26.8) | 123 (73.2) | 168 (49.6) | Female |
|  |  |  |  |  | **Disease duration** |
|  | Ref | 1 (10.0) | 9 (90.0) | 10 (3.0) | Minutes |
| 0.224 | 3.6 (0.45-29.4) | 60 (28.8) | 148 (71.2) | 60 (17.7) | 2-24 hours |
| 0.222 | 3.7 (0.45-30.4) | 35 (29.2) | 85 (70.8) | 35 (10.3) | 3 days |
|  |  |  |  |  | **Severity of disease** |
|  | Ref | 5 (3.7) | 129 (96.3) | **134 (39.5)** | Mild |
| <0.001 | 17.2 (6.7-44.0) | 76 (40.0) | 114 (60.0) | 190(56.0) | Moderate |
|  |  | 15 (100) | 0 | 15(4.5) | Severe |
|  |  |  |  |  | **Previous doctor's visit** |
|  | Ref | 74 (28.2) | 188 (71.8) | 262 (77.3) | No |
| 0.955 | 1.02 (0.58-1.78) | 22 (28.6) | 55 (71.4) | 77 (22.7) | Yes |
|  |  |  |  |  | **Number of times contacted the service** |
|  | Ref | 92 (27.7) | 240 (72.3) | 332 (98.0) | 1 time |
| 0.107 | 3.48 (0.76-15.8) | 4 (57.1) | 3 (42.9) | 7 (2.0) | Twice |
| 0.506 | 0.95 (0.81-1.11) | 3.2±1.9 | 3.3±1.4 | **3.3±1.6** | **Length of conversation** |
|  |  |  |  |  | **Correspondence to protocol** |
|  | Ref | 4 (22.2) | 14 (77.8) | **18 (5.3)** | No |
| 0.557 | 1.41 (0.45-4.4) | 92 (28.7) | 229 (71.3)) | **321 (94.7)** | Yes |

***** multi-variable logistic regression of ED referral [medical factors]; adjusted ORs
