# Supplementary material for: Assessing patient safety in a pediatric telemedicine setting: a multi-methods study
Source: BMC Med Inform Decis Mak. 2020 Apr 3;20:63. doi: 10.1186/s12911-020-1074-7 (PMC7126468; doi:10.1186/s12911-020-1074-7)
Supplement: Supplementary file 2 — Additional file 2: Supplemental Table 2. Medical Factors and Decision Reasonableness. [file 12911_2020_1074_MOESM2_ESM.docx]

**Supplemental Table 2: Medical Factors and Decision Reasonableness**

|  |  | **Reasonability** | |  |  |
| --- | --- | --- | --- | --- | --- |
| **p-value** | **OR 95%CI** | **Yes**  **N=27** | **Yes**  **N=312** | **Total**  **339** |  |
|  |  |  |  |  | **Age** |
|  | Ref | 13 (10.5) | 111 (89.5) | 124 (36.6) | <=1 |
| 0.110 | 0.46 (0.18-1.19) | 7 (5.1) | 130 (94.9) | 137 (40.4) | 1-5 |
| 0.458 | 0.61 (0.17-2.25) | 3 (6.7) | 42 (93.3) | 45 (13.3) | 5-10 |
| 0.788 | 1.18 (0.36-3.9) | 4 (12.1) | 29 (87.9) | 33 )9.7) | >10 |
|  |  |  |  |  | **Gender** |
| 0.879 | Ref | 14 (8.2) | 157 (91.8) | 171 (50.4) | Male |
|  | 0.94 (0.43-2.1) | 13 (7.7) | 155 (92.3) | 168 (49.6) | Female |
|  |  |  |  |  | **Disease duration** |
|  | Ref | 2 (20.0) | 8 (80.0) | 10 (3.0) | Minutes |
| 0.162 | 0.31 (0.06-1.6) | 15 (7.2) | 193 (92.8) | 60 (17.7) | 2-24 hours |
| 0.238 | 0.36 (0.07-1.95) | 10 (8.3) | 110 (91.7) | 35 (10.3) | 3 days |
|  |  |  |  |  | **Severity of disease** |
|  | Ref | 10 (7.5) | 124 (92.5) | **134 (39.5)** | Mild |
| 0.634 | 1.2(0.54-2.75) | 17 (8.9) | 173 (91.1) | 190(56.0) | Moderate |
|  |  | 0 | 15 (100) | 15(4.5) | Severe |
|  |  |  |  |  | **Previous doctor's visit** |
|  | Ref | 22 (8.4) | 240 (91.6) | 262 (77.3) | No |
| 0.589 | 0.76 (0.28-2.07) | 5 (6.5) | 72 (93.5) | 77 (22.7) | Yes |
|  |  |  |  |  | **Number of times contacted the service** |
|  |  | 27 (8.1) | 305 (91.9) | 332 (98.0) | 1 time |
|  |  | 0 | 7 (100)) | 7 (2.0) | Twice |
| 0.444 | 1.09 (0.88-1.35) | 3.5±2.7 | 3.3±1.4 | **3.3±1.6** | **Length of conversation** |
|  |  |  |  |  | **Correspondence to protocols** |
|  | Ref | 11 (61.1) | 7 (38.9) | **18 (5.3)** | No |
| <0.001 | 0.03 (0.01-0.09) | 16 (5.0) | 305 (95.0) | **321 (94.7)** | Yes |

***** Multi-variable logistic regression of reasonableness of decisions [medical factors];

adjusted ORs
