# Supplementary material for: Assessing patient safety in a pediatric telemedicine setting: a multi-methods study
Source: BMC Med Inform Decis Mak. 2020 Apr 3;20:63. doi: 10.1186/s12911-020-1074-7 (PMC7126468; doi:10.1186/s12911-020-1074-7)
Supplement: Supplementary file 3 — Additional file 3: Supplemental Table 3. Medical factors and Secondary Decisions. [file 12911_2020_1074_MOESM3_ESM.docx]

**Supplemental Table 3: Medical factors and Secondary Decisions***

|  |  | **Secondary Decision** | |  |  |
| --- | --- | --- | --- | --- | --- |
| **p-value** | **OR 95%CI** | ***Intervention/***  **clarification**  **N=62** | **Waiting**  **N=181** | **Total**  **243** |  |
|  |  |  |  |  | **Age** |
|  | Ref | 17 (17.7) | 79 (82.3) | 96 (39.5) | <=1 |
| 0.220 | 1.5 (0.77-3.1) | 24 (25.0) | 72(75.0) | 96 (39.5) | 1-5 |
| 0.007 | 3.3 (1.4-8.1) | 13 (41.9) | 18 (58.1) | 31 (12.8) | 5-10 |
| 0.033 | 3.1 (1.1-8.7) | 8 (40.0) | 12 (60.0) | 20 (8.2) | >10 |
|  |  |  |  |  | **Gender** |
| 0.012 | Ref | 22 (18.3) | 98 (81.7) | 120 (49.4) | Male |
|  | 2.1 (1.18-3.9) | 40 (32.5) | 83 (67.5) | 123 (50.6) | Female |
|  |  |  |  |  | **Disease duration** |
|  | Ref | 3 (33.3) | 6 (66.7) | 9 (3.7) | Minutes |
| 0.681 | 0.74 (0.18-3.1) | 40 (27.0) | 108 (73.0) | 148 (61.2) | 2-24 hours |
| 0.464 | 0.58 (0.13-2.5) | 19 (22.4) | 66 (77.6) | 85 (35.1) | 3 days |
|  |  |  |  |  | **Severity of disease** |
|  | Ref | 32 (24.8) | 97 (75.2) | **129 (53.1)** | Mild |
| 0.788 | 1.08(0.61-1.9) | 30 (26.3) | 84 (73.7) | 114(46.9) | Moderate |
|  |  |  |  |  | **Previous visit to doctor** |
|  | Ref | 54 (28.7) | 134 (71.3) | 188 (77.4) | No |
| 0.038 | 0.42 (0.19-0.95) | 8 (14.5) | 47 (85.5) | 55 (22.6) | Yes |
|  |  |  |  |  | **Number of times contacted the service** |
|  | Ref | 61 (25.4) | 179 (74.6) | 240 (98.0) | 1 time |
| 0.756 | 1.47 (0.13-16.5) | 1 (33.3) | 2 (66.7) | 3(2.0) | Twice |
| 0.057 | 1.21 (0.99-1.46) | 3.7±1.5 | 3.2±1.4 | **3.3±1.4** | **Length of conversation** |
|  |  |  |  |  | **Correspondence to protocol** |
|  | Ref | 3 (21.4) | 11 (78.6) | **14 (5.8)** | No |
| 0.7181 | 1.27 (0.34-4.7) | 59 (25.8) | 170 (74.2) | **229 (94.2)** | Yes |

* Multi-variable logistic regression of Secondary decisions [medical factors]; adjusted ORs

** *Active decisions*: Intervention – providing treatment (e.g., sending a digital prescription or other instructions) or determining that additional information is needed (follow-up consultation or video chat).
