# Supplementary material for: Assessing patient safety in a pediatric telemedicine setting: a multi-methods study
Source: BMC Med Inform Decis Mak. 2020 Apr 3;20:63. doi: 10.1186/s12911-020-1074-7 (PMC7126468; doi:10.1186/s12911-020-1074-7)
Supplement: Supplementary file 4 — Additional file 4 :Supplemental Table 4. Significant Non-medical factors- multivariate analyses. [file 12911_2020_1074_MOESM4_ESM.docx]

**Supplemental Table 4: Significant Non-medical factors- multivariate analyses ***

| **Decision Reasonableness** | | **Secondary Decision** | | **Primary Decision** | | **Factor** |
| --- | --- | --- | --- | --- | --- | --- |
| p-value | OR95%CI | p-value | OR95%CI | p-value | OR95%CI |  |
|  |  |  |  |  |  | **Place of residence** |
|  |  |  |  |  | Ref | Center |
|  |  |  |  | 0.901 | 1.05(0.52-2.1) | Periphery, south |
|  |  |  |  | <0.001 | 3.17 (1.7-5.9) | Periphery, north |
|  |  |  |  |  |  | **Doctor's gender** |
|  |  |  |  |  | Ref | Male |
|  |  |  |  | 0.024 | 1.9 (1.09-3.2) | Female |
|  |  |  |  |  |  | **Doctor's age** |
|  | Ref |  |  |  |  | 40-50 |
| 0.146 | 4.8 (0.58-40.6) |  |  |  |  | 50-60 |
| 0.010 | 15.0 (1.9-11.5) |  |  |  |  | >60 |
|  |  |  |  |  |  | **Doctor's specialty** |
|  |  |  |  | 0.029 | 2.95 (1.1-7.7) | Pediatrician |
|  |  |  |  |  | Ref | Sub-specialization |
|  |  |  |  |  |  | **Shared Decisions** |
|  | Ref |  | Ref |  | Ref | No |
| 0.010 | 4.00 (1.40-11.5) | <0.001 | 15.2(3.7-62.6) | <0.001 | 4.9 (2.2-11.05) | Yes |
|  |  |  |  |  |  | **Gender** |
|  |  |  | Ref |  |  | Male |
|  |  | 0.008 | 2.4 (1.26-4.67) |  |  | Female |
|  |  |  |  |  |  | **Contacting parent** |
|  |  |  | Ref |  |  | Mother |
|  |  | 0.027 | 2.8 (1.3-6.1) |  |  | Father |
|  |  |  |  |  |  | **Previous doctor's visit** |
|  |  | 0.018 | 2.9 (1.2-7.2) |  |  | No |
|  |  |  | Ref |  |  | Yes |

* Multi-variable logistic regression of primary decisions, secondary decisions, and decision reasonableness (Non-medical factors); adjusted ORs.
