# Supplementary material for: Assessing patient safety in a pediatric telemedicine setting: a multi-methods study
Source: BMC Med Inform Decis Mak. 2020 Apr 3;20:63. doi: 10.1186/s12911-020-1074-7 (PMC7126468; doi:10.1186/s12911-020-1074-7)
Supplement: Supplementary file 5 — Additional file 5: Supplemental Table 5. Quotes from Interviews with Physicians by Themes. [file 12911_2020_1074_MOESM5_ESM.docx]

**Supplemental Table 5**

**Quotes from Interviews with Physicians by Themes**

|  | **Quote** | **No. of Participant** |
| --- | --- | --- |
| **Theme 1: Intuition** | | |
| **1** | *"You learn to rely on intuition and on the parent – Your feelings as to whether there is someone who understands you or that in this case, your guidance will not help."* | 15 |
|  | *"I use intuition. Sometimes you feel that the parents are actually lying, trying to get what they want."* | 2 |
|  | *"For example, with head trauma, there is no room for intuition or judgment, especially with infants under the age of six months. I refer them directly to the emergency room... There are situations where a red flag is raised and you can also use your intuition, such as vomiting, abdominal pain, hemorrhagic rash, or restlessness that I cannot check closely. In this case, I tend to send the patient to the ED… In difficult situations, I ask a lot of questions to make sure there are no specific dangers (such as meningitis or dehydration). In such cases, I ask more detailed questions and provide more detailed guidelines."* | 1 |
|  | *"In addition to the child's medical condition, the parents also affect my decision. Some parents want to go to the emergency room and don't care about anything else. Sometimes I 'feel' (i.e., use my intuition) that the parents are afraid and do not trust themselves, so I refer them to the emergency room (because of their anxiety). In addition, if I feel that they may misunderstand me (due to language difficulties or a low level of understanding), it's easy for me to refer them to the ED."* | 12 |
|  | *"The bottom line is that my contribution was minor because I felt a great sense of responsibility; I was afraid that I would miss something because I could not physically see the patient. There is no specialization in telemedicine. On the one hand, you have a grave responsibility, but on the other hand you have limited tools and visibility. It's hard to make a decision that you can be at peace with, since the diagnosis relies on a lot of subjective things (such as your intuition and what the parents tell you)."* | 3 |
|  | *"There is adversity, especially regarding certain decisions – I am sometimes hesitant about what to do, since I'm alone, especially at nights, and have to rely a lot on my intuition. For instance, a child who swallowed a battery, the battery is then found on the table but it's leaking, but the parents don't want to go to the ED…"* | 9 |
| **Theme 2: Experience** | | |
| **2** | *"During my first few days on the job, I was afraid I would miss things or that there would be problems. After a while, I began to work with more confidence and less stress."* | 4 |
|  | *"There are some difficult aspects. At first, I felt insecure, but over time I gained experience (even the ability to diagnose better than the face-to-face doctor! (Like diagnosing a child with diabetic ketoacidosis) …*  *There is some difficulty in making decisions in this situation, but I feel relatively confident to make the decision - because I have experience in the field, even when I am not 100% sure…"* | 14 |
|  | *"Personally, I have no difficulty [providing telemedicine services], but you need experience. It can be dangerous because you are not 100% protected [from errors] in certain situations, such as meningococcemia, as you cannot physically examine the patient!*  *Personally, I was not too concerned, thanks to my professional experience, and if I wasn't certain, I would refer the patient to the emergency room (especially as no one is restricting us).*  *I feel that people appreciate the help I gave them. I did encounter unsatisfactory reviews. I always received positive feedback."* | 13 |
| **Theme 3: Rule of thumb and protocols** | | |
| **3** | *"For example, with head trauma, there is no room for intuition or judgment, especially with infants under the age of six months. I refer them directly to the emergency room... There are situations where a red flag is raised and you can also use your intuition, such as vomiting, abdominal pain, hemorrhagic rash, or restlessness that I cannot check closely. In this case, I tend to send the patient to the ED… In difficult situations, I ask a lot of questions to make sure there are no specific dangers (such as meningitis or dehydration). In such cases, I ask more detailed questions and provide more detailed guidelines."* | 1 |
|  | *"I use rules of thumb… as with babies less than a month old. There are also rules that I made for myself. For example, if I suspect dehydration as a complication of gastroenteritis, I send the child to the emergency room ... Sometimes, I call the parents back two hours later to check on the child."* | 4 |
|  | *"I use some rules of thumb. For example, if a young boy is able to jump around, then he does not have appendicitis… "* | 8 |
|  | *"I use protocols, mainly about head injuries with a loss of consciousness and vomiting."* | 2 |
|  | *"I use protocols. For example, head injuries among babies under the age of six months, or a high fever among babies younger than one month old. These make it easier to make a decision."* | 12 |
|  | *"An example of cognitive bias is when there is an exceptional case – after which everyone is much stricter with themselves. On the other hand, if someone complains that I sent too many patients to the emergency room, then I will reduce the number of referrals in the near future."* | 5 |
|  | *"I am aware of possible cognitive biases, and always think about the worst. Sometimes, we tend to relate to the caller's profession, which can be misleading. For example, once, a mother called me and she was a nurse. I prescribed her child with Ventolin for inhalation, but she thought the solution was to be administered by mouth instead. Finally, I referred her to the emergency room."* | 12 |
| **Theme 4: Shared decision-making with the parents** | | |
| **4** | *"Sometimes, I try to share my thoughts with the parents, offering a number of options. In other cases, however, I am unambiguous."* | 2 |
|  | *"I usually share, but I do not consult. I give my opinion and explain it, and only then do I wait for feedback."* | 3 |
|  | *"I used to share my decision-making process with the parents. If there are several options, then I let the parents decide. In such a case, I depend on them."* | 4 |
|  | *"I only try to share my thoughts in about 20% of the cases, and only when I feel that the parents understand me. After all, the parents know their child the best."* | 7 |
|  | *"You must share your decision with the parents. Nowadays, medicine has become Internet-based, with parents being much more aware…"* | 8 |
|  | *"Sometimes I offer several options and their consequences, and then let the parents decide."* | 9 |
|  | *"I rarely provide parents with options... I usually prefer to make my decision. I do offer options in some cases, for example, a child with a leg injury, no fracture, but a lot of pain..."* | 11 |
| **Theme 5: Considering non-medical factors** | | |
| **5** | *"In addition to medical factors, the parents' tone of voice and level of stress may affect my decision – even if it seems to be a simple diagnosis… Language is also a factor. For example, new immigrants (Ethiopians) do not always understand me and I therefore tend to send them to the ED…"* | 8 |
|  | *"What affects me the most is the child's medical condition, but I also consider impression of the parents – and their expectations. Some parents sound thoughtful and educated, and if the mother asks for a referral to the ED, there is a chance I will give it to her – providing I get the impression she will use the referral logically. However, if the parents have difficulty understanding me, I am more likely to refer the patient to the ER."* | 11 |
|  | *"In addition to the child's medical condition, the parents affect my decision. Some parents want to go to the emergency room and don't care about anything else. Sometimes I feel that the parents are afraid and don't trust themselves, so I refer them to the emergency room. In addition, if I feel that they have misunderstood me (due to language difficulties or a low level of understanding), I am more prone to refer them to the ED."* | 12 |
|  | *"You have to trust the parents' information and rely on them to follow the instructions correctly. If I feel that the chances of me being understood are slim (due to a lack of understanding or over-sophistication on the part of the parents) – I will refer them to the ED more easily."* | 1 |
|  | *"Lack of understanding or cooperation by the parents is also a factor. I should get a feeling that on the other end of the phone there is someone I can rely on. If the parents aren't fluent in Hebrew, I will attempt to pass them to a different doctor who speaks Arabic or Russian, for example). If there is no choice, I will send them to the ED."* | 15 |
|  | *"The first thing is my impression of the parents' understanding. If I get the impression that they won't implement my instructions correctly, I will be more prone to refer them to the ED."* | 1 |
|  | *"There are many other things that affect me besides the patient's clinical condition. For example, what the parents say and how they speak. Sometimes they are over-dramatic, in which case I specifically don’t refer to the ED."* | 8 |
|  | *"I am mainly affected by medical (clinical) factors, but also by other factors, such as the parents' stress, my uncertainty in their ability to treat the child, their explicit request to go to the ED, or the impression that they are not competent enough."* | 10 |
|  | *"As we do not really know the family, we have to use additional clues. For example, if we get the impression that they will not successfully treat their child at home, I will refer them to the emergency room."* | 13 |
|  | *"When I do not refer the patient to the ED, I explain to the parents exactly what to do, and remind them that they can call the center again later, or go to an emergency medical center in the community. The parents' literacy also affects me: I must be sure that he understands and is comfortable with my decision. If the parent feels uncomfortable -I change the decision."* | 8 |
|  | *"It's very important that the parents understand the problem and the medical explanation (i.e., taking care of the patient at home, going to the doctor the following day, calling the online service again, etc.) The parent's ability to function and cope if the symptoms worsen is also important, such as a mother who will not be able to cope even though she seems to understand my instructions."* | 9 |
|  | *"Aside from the medical condition, the patient's place of residency is also important. Living far from a medical care facility is a factor, and I will be more likely to consider an ED referral for such a patient. I will start by asking more questions about the availability of the doctor nearby."* | 14 |
|  | *"Another thing to consider is the availability of nearby medical services. As such, if access is to a medical services is insufficient in the case of worsening symptoms (such as living over an hour away from any relevant services), I will prefer to send them to the ED.*  *The same goes for weekends and holidays, when clinics will not be open the following day. In addition, I may refer patients to the ED if their parents specifically request this or are already on their way to the ED."* | 1 |
|  | *"If the family lives far away or if it's the weekend and there is no doctor the following day, then I will try to persuade the parents to go to an emergency medical center or ED."* | 7 |
| **Theme 6: Additional tools** | | |
| **6** | *"If I needed additional information, I would arrange a video call or a follow-up call at a later time. Rarely would I consult with a senior physician."* | 1 |
|  | *"I would not usually consult with the senior physician. I usually start a video conversation... despite technical faults that get in the way. If that doesn't work, I'll ask for digital pictures. Sometimes, I would tell the parents that I will get back to them at a later time."* | 6 |
|  | *"I suggest sending photos via email, especially for skin lesions. Sometimes it's better than a video call."* | 1 |
|  | *"Despite the difficulty making an online decision, digital pictures and videos often compensate for the lack of physical examination. In one case, I managed to correctly diagnose a child with intussusception!"* | 5 |
|  | *"It is important to have a senior doctor on call. It helps in situations of indecision. In addition, it should be possible to receive pictures by e-mail from the parents, which is especially effective with rashes. Video calls may be tiresome for patients, but it is often worth it. Moreover, training doctors for online work is effective because it focuses you. Official protocols are also helpful."* | 4 |
